# Metformin Is Associated with Reduced Tissue Factor Procoagulant Activity in Patients with Poorly Controlled Diabetes

**DOI:** 10.1007/s10557-020-07040-7

**Published:** 2020-09-17

**Authors:** Marco Witkowski, Julian Friebel, Termeh Tabaraie, Sinah Grabitz, Andrea Dörner, Lena Taghipour, Kai Jakobs, Bernd Stratmann, Diethelm Tschoepe, Ulf Landmesser, Ursula Rauch

**Affiliations:** 1 Charité Centrum 11, Department of Cardiology, Charité – Universitätsmedizin Berlin, Campus Benjamin Franklin, Hindenburgdamm 30, 12200 Berlin, Germany; 2 Herz und Diabeteszentrum NRW, Ruhr Universität Bochum, Bad Oeynhausen, Germany

**Keywords:** Metformin, diabetes mellitus, coagulation, tissue factor, vascular inflammation, thrombosis, microRNA, vascular complications, cardiovascular disease

## Abstract

**Purpose:**

Metformin is the first-line antidiabetic drug and shown to reduce cardiovascular risk independent from its glucose lowering action. Particularly in poorly controlled diabetes, tissue factor (TF) is expressed in the vasculature and accounts for thromboembolic complications. Here, we aimed to assess the effect of metformin on TF activity and markers of vascular inflammation in poorly controlled type 2 diabetes.

**Methods:**

In a cohort of patients with uncontrolled type 2 diabetes (glycosylated hemoglobin 8.39 *±* 0.24%, 68.1 *±* 2.6 mmol/mol, *n* = 46) of whom half of the individuals were treated with metformin and the other half did not receive metformin as part of an anti-diabetic combination therapy, we assessed TF activity and markers of vascular inflammation. In vitro, human monocytic cells (THP-1) were exposed to metformin and TF expression measured in the presence and absence of the AMP-activated protein kinase (AMPK) activator 5-aminoimidazole-4-carboxamide riboside (AICAR) or the AMPK inhibitor compound C.

**Results:**

In the patients, metformin treatment was associated with lower levels of TF protein (241.5 ± 19 vs. 315.4 ± 25 pg/mL, *p* = 0.03) and reduced TF activity (408.9 ± 49 vs. 643.8 ± 47 U/mL, *p* = 0.001) compared with controls. Moreover, the patients on metformin showed lower levels of vascular cell adhesion molecule (VCAM)1 (26.6 ± 1.4 vs. 35.03 ± 3.1 ng/mL, *p* = 0.014) and higher expression of miR-126-3p/U6sno (11.39 ± 2.8 vs. 4.26 ± 0.9, *p* = 0.006), a known post-transcriptional down regulator of TF and VCAM1. In vitro, metformin dose-dependently reduced lipopolysaccharide (LPS)-induced TF expression in THP-1 cells. The AMPK activator AICAR alone lowered TF expression in THP-1, while the AMPK inhibitor compound C abrogated the metformin-dependent reduction in TF expression.

**Conclusions:**

Our data are the first to report that metformin is associated with reduced plasma TF procoagulant activity possibly explaining—at least in part—the vasculoprotective properties of metformin.

**Electronic supplementary material:**

The online version of this article (10.1007/s10557-020-07040-7) contains supplementary material, which is available to authorized users.

## Introduction

Being prescribed annually for more than 120 million patients worldwide, metformin is the first-line antidiabetic drug for patients with type 2 diabetes mellitus. Activation of AMP-activated protein kinase (AMPK) that involves the AXIN/LKB1-v-ATPase-Ragulator pathway by metformin reduces hepatic glucose production and improves peripheral insulin sensitivity [1]. Most importantly, metformin reduces cardiovascular outcomes independent from its glucose lowering effect [2]. This unique feature prompted in vitro and in vivo discovery of pleiotropic vascular effects, including control of endothelial inflammation [3], anti-thrombotic properties, effects mediated by gut microbiota, and impact on microRNA (miR) synthesis [4, 5] pointing to additional epigenetic effects.

Individuals with poorly controlled diabetes have high levels of blood-borne tissue factor (TF), the primary initiator of clotting, leading to Factor(F)Xa generation, inflammatory signaling and vascular complications, the leading cause for mortality in diabetes [6–9]. The effect of metformin on TF activity in the clinical setting of advanced diabetes remains unclear. Here, we sought to assess the effect of metformin on TF activity and vascular inflammation in poorly controlled type 2 diabetes.

## Methods

The study protocol was approved by the local ethics committee and was in accordance with the ethics principles in the Declaration of Helsinki. Prior to participation, each patient gave a written informed consent. Patients (*n* = 46) admitted to a diabetes center for poor glycemic control (glycosylated hemoglobin (HbA1c) of > 7.5% and/or unstable glycemic control with recurrent hypoglycemia) in the absence of an acute illness or recent (< 6 month) acute cardiovascular events (myocardial infarction, stroke, or transient ischemic attack) were enrolled into the study as described previously [10]. At the time of admission to the hospital, half of the patients was already treated with metformin for at least 6 month at a stable dose adjusted to renal function as part of an anti-diabetic combination therapy (metformin group, *n* = 23) and the other half of the patients did not receive metformin (control group, *n* = 23). All patients received comprehensive education for disease management including diet. Table 1 shows the patient characteristics. Peripheral blood was obtained by venipuncture into heparinized, citrate, or EDTA tubes, and markers for coagulation (TF protein and FXa generation in plasma) and inflammation (including leukocyte count, neutrophils, myeloperoxidase, c-reactive protein (CRP), VCAM-1, low-density lipoprotein (LDL) cholesterol, and fibrinogen) were assessed on admission.

**Table 1:** Patient characteristics of both groups

Characteristics	Control *n* = 23	Metformin *n* = 23	*P* value
Sex (female)	7	5	0.738
Age (years)	65.2 ± 2.0	64.1 ± 1.8	0.604
History of stroke (%)	21.7	0	0.049*
History of MI (%)	21.7	8.6	0.362
CAD (%)	43.4	39.1	> 0.999
Hypertension (%)	100.0	100.0	> 0.999
PAD (%)	30.4	21.7	0.738
BMI (kg/m^2^)	31.0 ± 1.1	33.6 ± 1.8	0.362
Smoking (%)	8.7	8.7	> 0.999
Neutrophils (%)	61.4 (55–67)	57.3 (46–64)	0.089
LDL cholesterol (mg/dL)	114.6 ± 7.9	110.9 ± 8.7	0.402
HDL cholesterol (mg/dL)	42.1 ± 2.2	43.3 ± 3.2	0.948
Total cholesterol (mg/dL)	182.3 ± 9.1	203.0 ± 11.3	0.162
Fibrinogen (mg/dL)	399.0 ± 27.2	345.4 ± 18.9	0.228
Myeloperoxidase (ng/mL)	16.4 ± 2.6	8.8 ± 0.9	0.093
CRP (mg/dl)	0.4 (0.15–1.2)	0.4 (0.23–0.63)	0.883
GFR (mL/min)	56.0 (45–80)	66.0 (58–87)	0.339
HbA1c (%)	8.3 ± 0.3	8.4 ± 0.3	0.866
HbA1c (mmol/mol)	67.7 ± 3.5	68.5 ± 3.8	0.900
Fasting blood glucose (mg/dl)	154.3 ± 12.7	146.2 ± 9.1	0.806
Medication			
insulin (%)	82.6	60.8	0.189
Sulfonylurea (%)	13.0	17.3	> 0.999
Acarbose (%)	13.0	8.6	> 0.999
Glinides (%)	4.3	13.0	0.607

*n* = 46; values presented are means ± SEM, medians (interquartile range) or percentages

*BMI* body mass index, *CAD* coronary artery disease, *CRP* c-reactive protein, *GFR* glomerular filtration rate, *HbA1c* glycated hemoglobin, *HDL* high-density lipoprotein, *LDL* low-density lipoprotein, *MI* myocardial infarction, *PAD* peripheral artery disease

* *p* < 0.05

## Cell Culture

Human monocytic THP-1 cells (purchased from ATCC) were grown in RPMI 1640 medium (Gibco) supplemented with 10% FBS and 1% penicillin/streptomycin (PAA). The cells were incubated in either 10 or 100 μM metformin for 24 h and stimulated with lipopolysaccharide (LPS) (10 μg/mL) for 2 h to induce TF expression. In some experiments the AMPK activator 5-aminoimidazole-4-carboxamide riboside (AICAR) (100 μM) or the AMPK inhibitor compound C (10 μM) were added to the medium for 24 h before LPS treatment. Human microvascular endothelial cells (HMEC-1, purchased from ATCC) were cultured in MCBD 131 medium (Gibco) supplemented with 10% FBS (Gibco), 100 U/mL penicillin/streptomycin (PAA), 2 mM l-glutamin (PAA), and 0.05 mg/mL hydrocortison. HMEC-1 were left untreated or stimulated with metformin at 1, 10, or 100 μM for 72 h and then stimulated with 10 ng/mL tumor necrosis factor(TNF)α prior to RNA extraction.

## ELISA Experiments and TF Activity

Specific ELISA systems were used to measure plasma concentrations of TF (Sekisui Diagnostics), VCAM-1 (R&D Systems), and myeloperoxidase (Abcam). The measurement of bona fide TF activity was performed as described before [11, 12]. The recombinant FVIIa was kindly provided by Novo Nordisk.

## Real-Time PCR

For real-time PCR, total mRNA was isolated with peqGOLD Trifast (Peqlab) for cell culture experiments or using the mirVana Paris Kit (Life Technologies) for patient blood plasma. Gene expression of TF (custom assay), VCAM1 (Hs01003372_m1), interleukin(IL)-1β (Hs01555410_m1), and IL-6 (Hs00174131_m1) was determined using TaqMan® assay (life Technologies) and normalized to Glyceraldehyde 3-phosphate dehydrogenase (GAPDH, Hs99999905_m1). miR-126 expression was analyzed via TaqMan® (hsa-miR-126-3p0002228) and normalized to U6 snRNA (001973).

## Statistics

The statistical analyses were performed using the software GraphPad Prism 7. Cell culture experiments were performed at least 3 times. Differences between 2 groups were calculated via Student’s *t* test or Mann–Whitney test depending on normality. For comparisons of 1 parameter between more than two groups an ANOVA test with Tukey’s post hoc test was used. For data sets with 4 groups and 2 variables a 2-way ANOVA test with Dunnett’s multiple comparison post hoc test was performed. Data are represented as mean ± SEM. *P* < 0.05 are considered statistically significant.

## Results

The individuals had a poorly controlled type 2 diabetes (defined as HbA1c of > 7.5% and/or unstable glycemic control with recurrent hypoglycemia, mean HbA1c 8.39 *±* 0.24%, 68.1 *±* 2.6 mmol/mol), and a prothrombotic state (mean FXa generation 526.4 *±* 38.2 U/mL). The patients presented a cardiovascular risk profile including obesity, hypertension, coronary artery disease, and history of thromboembolic complications in some cases. Except from a greater history of stroke in the control group, there were no significant differences in age, BMI, fasting blood glucose, renal function, or other anti-diabetic drugs between the groups (Table 1).

**Fig. 1:**
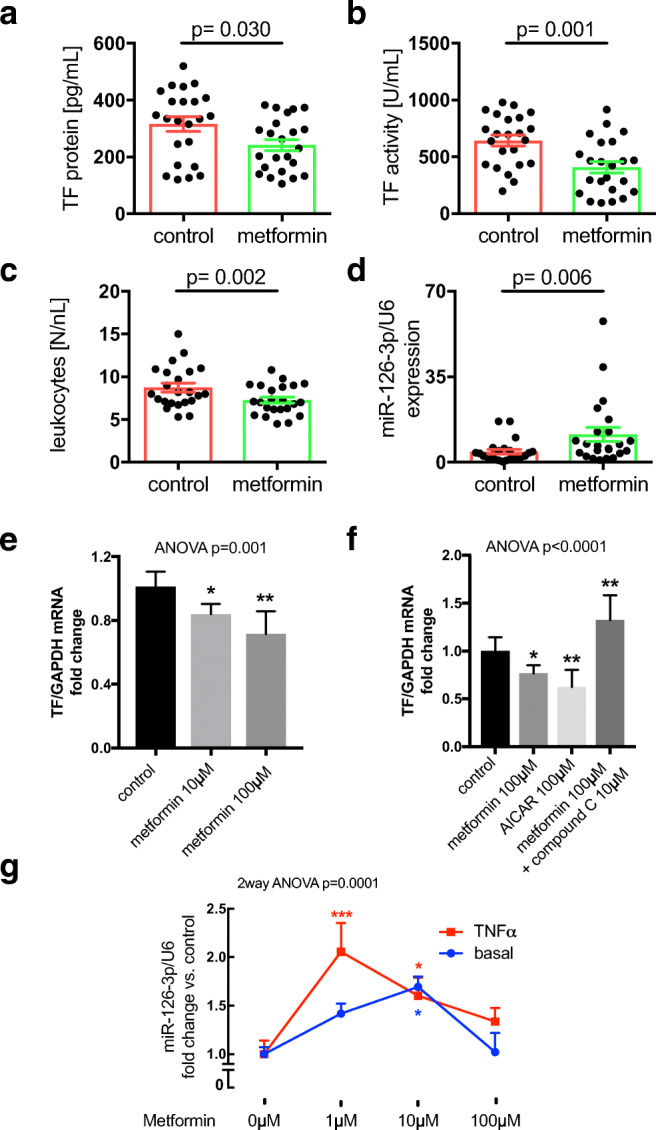
Metformin reduces tissue factor procoagulant activity in diabetes. Patient cohort: Plasma of patients with diabetes receiving metformin or not was analyzed with respect to TF protein (**A**), TF activity (**B**), leukocyte count (**C**), and miR-126 expression (**D**). *n* = 46; shown are mean ± SEM; differences between groups were measured by a Student’s *t* test or Mann–Whitney test. *P*-values are indicated. In vitro experiments: THP-1 cells were left untreated or incubated with the indicated concentrations of metformin for 24 h and then induced with 10 μg/mL LPS for 2 h and TF mRNA expression assessed (**E**). THP-1 cells were cultured in the absence or presence of metformin, AICAR, or metformin together with compound C at the indicated concentrations for 24 h. The cells were then stimulated with 10 μg/mL LPS for 2 h and TF mRNA quantified (**F**). HMEC-1 were left untreated or exposed to different concentrations of metformin as indicated for 72 h. miR-126 expression was then assessed under basal conditions or following stimulation with TNFα for 2 h (**G**). *n* ≥ 5; groups were compared by ANOVA with Tukey’s post hoc test (**E**,**F**) or 2-way ANOVA with Dunnett’s multiple comparison post hoc test (**G**). *p < 0.05, **p < 0.01, ***p < 0.0001 vs. control

Metformin treatment was associated with reduced TF protein (241.5 ± 19.4 vs. 315.4 ± 25.7 pg/mL) and TF activity (408.9 ± 49.9 vs. 643.8 ± 47.2 U/mL) in the blood (Fig. 1 A and B) as well as reduced markers of systemic and vascular inflammation evidenced by leukocytes (7.2 ± 0.3 vs. 8.7 ± 0.5 N/nL; Fig. 1C) and VCAM1 (26.6 ± 1.4 vs. 35.03 ± 3.1 ng/mL, *p* = 0.014, not shown). Metformin also tended to be related to lower neutrophils and myeloperoxidase (Table 1). Surprisingly, in this setting of advanced state of the disease, both groups had the same HbA1c suggesting that the reduction in thrombogenicity is independent of glycemic control. Moreover, patients on metformin exhibited higher expression of miR-126-3p (11.39 ± 2.8 vs. 4.26 ± 0.9, Fig. 1D), a known post-transcriptional down regulator of TF and VCAM1 mRNA [12]. In vitro, metformin dose-dependently reduced LPS-induced TF expression in human monocytic cells (THP-1) (Fig. 1E). This effect was phenocopied by the AMPK activator AICAR alone. In contrast, the AMPK inhibitor compound C abrogated the metformin-dependent reduction in TF expression in THP-1 (Fig. 1F). Metformin also reduced the expression of the pro-inflammatory cytokine IL-1β (but not IL-6) in LPS-induced THP-1, which was blunted in the presence of compound C (suppl. Figure 1A,B). Finally, exposure of HMEC-1 to metformin resulted in an increase in miR-126 expression, which was even more pronounced in the presence of the inflammatory cytokine TNFα (Fig. 1G).

## Discussion

Here, we show for the first time that metformin treatment is associated with a reduced TF-depending pro-thrombotic state in poorly controlled diabetes. Previous studies enrolling patients with a better controlled type 2 diabetes (HbA1c 6.48 ± 0.65%) reported that metformin led to a moderate reduction in blood TF activity in some individuals [13]. Our patients showed an advanced state of the disease with high glycemic indices that are known to promote pro-inflammatory signaling that contributes to vascular TF expression. The pro-thrombotic state in our patient cohort allowed us to capture metformin’s anticoagulant properties that may not be apparent in well-controlled type 2 diabetes.

Interestingly, we found no differences in blood glucose but lower levels of leukocytes and VCAM1 in patients treated with metformin. This highlights that in poorly controlled diabetes metformin exerts anti-thrombotic effects beyond glycemic control most likely through modulation of vascular inflammation. In line, metformin treatment was also found to be associated with reduced TF-positive microvesicles in the blood of patients with polycystic ovary syndrome, a disease that involves inflammatory signaling [14].

Our hypothesis is supported by studies showing the anti-inflammatory properties of metformin. In vitro, vascular wall, and blood cells exposed to metformin exhibit reduced IL-1β and LPS-induced activation of nuclear factor kappa B [3] and early growth response (egr)-1, respectively, both transcription factors drive vascular TF transcription. We found metformin to reduce LPS-induced monocytic expression of the inflammatory cytokine IL-1β but not IL-6. Another study in patients with impaired glucose tolerance treated with simvastatin reported that addition of metformin reduced both IL-1β and IL-6 in LPS-treated peripheral blood monocytes [15]. The very limited LPS-induced IL-6 release in THP-1 compared with human monocytes [16] may explain that we did not observe changes in IL-6 expression in THP-1 exposed to metformin. Interestingly, our patients on metformin showed a trend towards a lower neutrophil count and reduced activity of myeloperoxidase, which has been implicated in endothelial TF production [17].

Most importantly, we confirmed that metformin inhibits LPS-induced TF expression in monocytes, the main source for TF activity in the blood, and provide novel evidence that this anticoagulant effect is mediated by the metformin target AMPK (Fig. 1E, F).

We have previously shown that miRs, such as mir-126 or miR19a, participate in the post-transcriptional control of TF activity and vascular inflammation in diabetes [12, 18]. Interestingly, metformin was also related to higher expression of the endothelial miR-126-3p in our patients (but not miR-181a that does not target the TF transcript, data not shown). We found that metformin exposure of HMEC-1 was sufficient to induce miR-126-3p expression under basal and inflammatory conditions (Fig. 1G). In line, recent mechanistic studies identified DICER, the key miR processing enzyme, to be upregulated by metformin resulting in expression of specific miRs [4]. Inhibition of some of those candidate miRs impaired the ability of metformin to reduce LPS-induced TNFα release in mouse macrophages [19]. These studies indicate that the vasculoprotective effects of metformin are also mediated by miRs. Our work provides clinical and experimental evidence that metformin may not only impact TF transcription via control of inflammatory transcription factors but also employs additional epigenetic control of thrombogenicity and vascular inflammation via altering circulating miRs. However, in our cross-sectional analysis in patients with type 2 diabetes that have already been treated with metformin for a long time, we cannot rule out differences in TF activity at baseline for each individual prior to the start of metformin as a potential confounder for our findings.

## Conclusion

Our data are the first to report that metformin is associated with reduced plasma TF procoagulant activity in patients with uncontrolled diabetes possibly involving AMPK- and miR-dependent control of vascular inflammation. Particularly, patients with poorly controlled diabetes and the presence of cardiovascular disease that have a high thrombotic risk may benefit from the anti-thrombogenic effects of metformin to prevent cardiovascular complications.

## Electronic supplementary material

ESM 1 (DOCX 61 kb)
