# Supplementary material for: Metformin Is Associated with Reduced Tissue Factor Procoagulant Activity in Patients with Poorly Controlled Diabetes
Source: Cardiovasc Drugs Ther. 2020 Sep 17;35(4):809–13. doi: 10.1007/s10557-020-07040-7 (PMC8266708; doi:10.1007/s10557-020-07040-7)
Supplement: Supplementary file 1 — (DOCX 61 kb) [file 10557_2020_7040_MOESM1_ESM.docx]

**Supplemental figure 1**: **Metformin reduces interleukin-1β in THP-1**

THP-1 cells were left untreated or incubated with the indicated concentrations of metformin or metformin together with compound C for 24h. The cells were then induced with 10µg/mL LPS for 2h and mRNA expression of IL-1β (A) and IL-6 assessed (B). n≥5, groups were compared by ANOVA with Tukey’s post hoc test *p<0.05, **p<0.01, ***p<0.0001 vs. control.
